# Plasmonic piezoelectric nanomechanical resonator for spectrally selective infrared sensing

**DOI:** 10.1038/ncomms11249

**Published:** 2016-04-15

**Authors:** Yu Hui, Juan Sebastian Gomez-Diaz, Zhenyun Qian, Andrea Alù, Matteo Rinaldi

**Affiliations:** 1 Department of Electrical & Computer Engineering at Northeastern University, 360 Huntington Avenue, Boston, Massachusetts 02115, USA; 2 Department of Electrical & Computer Engineering at The University of Texas at Austin, 1616 Guadalupe St., UTA 7.215, Austin, Texas 78701, USA

## Abstract

Ultrathin plasmonic metasurfaces have proven their ability to control and manipulate light at unprecedented levels, leading to exciting optical functionalities and applications. Although to date metasurfaces have mainly been investigated from an electromagnetic perspective, their ultrathin nature may also provide novel and useful mechanical properties. Here we propose a thin piezoelectric plasmonic metasurface forming the resonant body of a nanomechanical resonator with simultaneously tailored optical and electromechanical properties. We experimentally demonstrate that it is possible to achieve high thermomechanical coupling between electromagnetic and mechanical resonances in a single ultrathin piezoelectric nanoplate. The combination of nanoplasmonic and piezoelectric resonances allows the proposed device to selectively detect long-wavelength infrared radiation with unprecedented electromechanical performance and thermal capabilities. These attributes lead to the demonstration of a fast, high-resolution, uncooled infrared detector with ∼80% absorption for an optimized spectral bandwidth centered around 8.8 μm.

## Abstract (web summary)

Plasmonic metasurfaces can provide exciting optical functionalities. Here, Hui *et al*. demonstrate an infrared sensor by combining plasmonic and piezoelectric electromechanical resonances, demonstrating efficient transduction of vibration with a strong and polarization-independent absorption over an ultrathin thickness.

## Graphical abstract

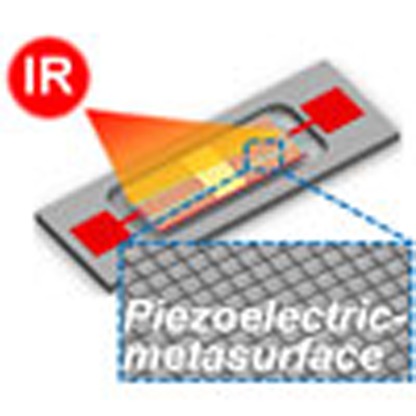

Infrared detector technologies were originally developed primarily for military demands, such as night vision, missile tracking, target acquisition and surveillance. In the past few decades, the use of infrared technologies for civilian applications has been steadily growing. Nowadays, infrared detectors can be found in a wide variety of applications, including medical diagnostics, biological and chemical threat detection, electrical power system inspection, infrared spectroscopy, and thermal imaging. Photon detection and thermal sensing are the two main approaches used for the implementation of infrared detectors. Photonic detectors exploit the interaction between photons and electrons in a semiconductor material to produce an electrical output signal upon exposure to infrared radiation^1^,^2^. They have the advantages of high signal-to-noise ratio (hence high resolution) and fast response time. However, to achieve such a high performance, they typically need cryogenic cooling to prevent thermally generated carriers^3^, making them bulky, expensive and power inefficient. On the other hand, thermal detectors rely on the temperature-induced change of material physical properties upon exposure to infrared radiation. They are generally less expensive, more compact and power efficient than semiconductor photon detectors, given their intrinsic capability to operate at room temperature, but they exhibit relatively worse resolution and slower response time. Several uncooled thermal detector technologies have been demonstrated including bolometers^4^, thermopiles^5^, pyroelectric detectors^6^ and more recently, resonant detectors (electromechanical^7^ and optical^8^).

Recently, the development of miniaturized, ultra-low-power and low-cost sensor technologies (including uncooled thermal detectors) have attracted great attention for the implementation of highly distributed wireless sensor networks, such as the internet of things, in which physical and virtual object are connected together through the exploitation of sensing and wireless communication functionalities. In this context, micro- and nanoelectromechanical systems (MEMS/NEMS) can have a tremendous impact, since they can simultaneously provide multiple sensing and wireless communication functionalities integrated in a small footprint. The use of MEMS/NEMS has been explored in a large number of applications, spanning from semiconductor-based technology^9^ to fundamental science^10^. In particular, MEMS technology has also been employed successfully for miniaturized and ultra-sensitive uncooled infrared detectors such as microbolometers^11^ and micromachined thermopile^12^. Among different MEMS/NEMS sensor technologies, the one based on a resonant-sensing mechanism offers significant advantages over other non-resonant approaches. In general, micro–nanoresonant sensors are characterized by a unique combination of high sensitivity to external perturbations, due to the greatly reduced dimensions of sensing element, and ultra-low-noise performance, due to the intrinsically high quality factor (*Q*) of such resonant systems. Furthermore, resonant sensors use frequency as the output variable, which is one of the physical quantities that can be monitored with the highest accuracy and converted to digital form by simply measuring zero crossings. Among all types of resonant sensors, the one based on MEMS/NEMS resonators can typically deliver the most compact and power efficient sensing solutions thanks to the use of on-chip transduction techniques (such as piezoelectric, electrostatic or thermal) in contrast with the bulky and off-chip optical actuation and readout techniques (requiring the use of power hungry lasers and other bulky optical components and interconnects) typically employed in optical resonators^8^,^13^ or optomechanical resonant systems^14^, which are not suitable for many low-power portable applications. The current bottleneck in the development of high-performance MEMS/NEMS resonant infrared detectors is the lack of deeply subwavelength and highly absorbing materials compatible with standard microfabrication processes and efficient transduction techniques. Indeed, the conventional approach to enable infrared absorptance in MEMS/NEMS resonant structures involves the integration of an infrared absorber (that is, a thin layer of lossy dielectric^8^,^15^,^16^ or a metal–insulator–metal grating^17^) on top of the vibrating body of the resonant transducer. Although a relatively weak infrared absorptance (<50% and polarization dependent) is typically achieved using this conventional approach, the electromechanical and thermal properties of the resonator (hence detection capability and power efficiency of the infrared sensor) are severely deteriorated due to the electrical and mechanical loading effects of the relatively bulky infrared absorbing material stack attached to the vibrating body of the micro/nanostructure^17^.

The fundamental challenge associated with efficient light concentration in planar structures with deeply subwavelength thickness has recently been addressed in the field of nanoplasmonics^18^,^19^,^20^. Metasurfaces with optical properties not found in nature have been synthesized by tailoring plasmonic resonances sustained by arrays of nanostructures with subwavelength dimensions. These effects have been utilized in a wide range of applications, including beam steering^21^,^22^, ultrathin focusing or diverging lenses^23^,^24^, reflectors^25^ and absorbers^26^,^27^. Thanks to these findings, plasmonically enhanced MEMS/NEMS has emerged as a promising research direction towards the development of miniaturized transducers able to convert electromagnetic energy into electric signals by simultaneously exploiting plasmonic, thermal and electromechanical properties^28^,^29^,^30^,^31^. In particular, uncooled infrared sensors consisting of a plasmonic absorber attached to a conventional MEMS thermal detector (that is, thermopile or microbolometer) have been demonstrated^32^,^33^, showing enhanced responsivity at specific wavelengths of interest in the infrared range. More recently, the integration of bulk metamaterials and nanoplasmonic infrared/THz absorbers in beam-type nanomechanical structures has also been demonstrated^34^. It has been shown that the plasmonically enhanced light absorption can induce a substantial beam deflection through the intrinsically high thermomechanical coupling of such free-standing nanomechanical structures. Even though these devices exhibit improved detection capabilities compared with some conventional MEMS-based thermal detectors, they require the use of relatively cumbersome and complex off-chip optical readouts to monitor the thermally induced deformation of the nanobeam. In this perspective, the achievement of efficient actuation and sensing of mechanical vibration in a plasmonic nanomechanical structure with intrinsically enhanced light absorption capability (without the need of integrating additional infrared absorbing materials) is highly desirable, being able to combine high sensitivity with power efficient on-chip transduction and readout.

Here we propose an ultrathin (650 nm) piezoelectric plasmonic metasurface forming the vibrating body of a nanomechanical resonator with unprecedented optical and electromechanical performance. By combining plasmonic and piezoelectric electromechanical resonances, we demonstrate efficient transduction of vibration in a nanomechanical structure with a strong and polarization-independent absorption coefficient over an ultrathin thickness, addressing all fundamental challenges associated with the development of performing resonant infrared detectors.

## Results

### Device design

**Figure 1:**
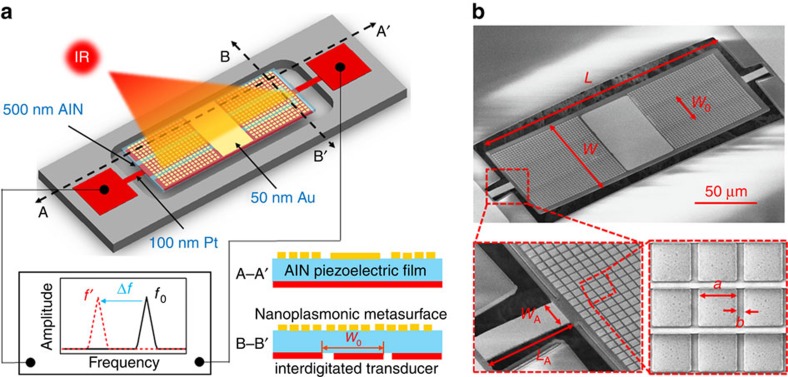
Overview of the plasmonic piezoelectric nanomechanical resonant infrared detector. (**a**) Mock-up view: an aluminum nitride nanoplate is sandwiched between a bottom metallic interdigitated electrode and a top nanoplasmonic metasurface. The incident IR radiation is selectively absorbed by the plasmonic metasurface and heats up the resonator, shifting its resonance frequency from *f*_0_ to *f*′ due to the temperature dependence of its resonance frequency. (**b**) Scanning electron microscopy images of the fabricated resonator, metallic anchors and nanoplasmonic metasurface. The dimensions of the resonator are as follows: *L*=200 μm; *W*=75 μm; *W*_0_=25 μm (19+6 μm); *L*_A_=20 μm; *W*_A_=6.5 μm. The dimensions of the unit cell of the plasmonic metasurface are as follows: *a*=1635, nm; *b*=310 nm. IR, infrared.

The proposed plasmonic piezoelectric NEMS resonator, illustrated in Fig. 1, is composed of an aluminum nitride (AlN) piezoelectric nanoplate (500-nm-thick) sandwiched between the two metal layers (Supplementary Fig. 1). The bottom layer (100-nm-thick platinum (Pt)) is patterned to form an interdigitated transducer (IDT) used to actuate and sense a high-order lateral-extensional mode of vibration in the nanoplate^35^; the top electrically floating layer (50-nm-thick gold) is patterned with the goal of confining the electric field induced by the bottom IDT across the piezoelectric nanoplate (Supplementary Fig. 2), while simultaneously enabling absorption of infrared radiation in the ultrathin piezoelectric nanoplate thanks to suitably tailored plasmonic resonances (Supplementary Fig. 3). The nanoplate is released from the silicon (Si) substrate to vibrate freely, and it is mechanically supported by two ultrathin Pt tethers (100-nm-thick, 6.5-μm-wide and 20-μm-long), which also provide electrical contact^36^. Such Pt tethers greatly improve the thermal isolation between the nanoplate and the Si substrate compared with conventional ones composed of an AlN–Pt stack^37^. The mechanical resonance frequency of the plasmonic piezoelectric resonator is defined by the equivalent Young's modulus *E*_eq_ and density *ρ*_eq_ of the resonant material stack, and the pitch of the interdigitated electrode *W*_0_ (electrode width plus spacing, see Fig. 1a), given by 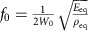. When an a.c. signal is applied to the bottom IDT of the device, the top electrically floating electrode acts to confine the electric field across the device thickness, and a high-order contour-extensional vibration mode is excited through the equivalent *d*_31_ piezoelectric coefficient of AlN^35^ when the frequency of the a.c. signal coincides with the natural resonance frequency, *f*_0_, of the resonator (Supplementary Note 1). If an infrared beam impinges on the device from the top (Fig. 1a), it is selectively absorbed by the metasurface, leading to a large and fast increase of the device temperature Δ*T*, due to the excellent thermal isolation and extremely low-thermal mass of the free-standing nanomechanical structure. Such infrared-induced temperature rise results in a shift in the mechanical resonance frequency of the resonator (from *f*_0_ to *f*_0_−Δ*f*) due to the intrinsically large temperature coefficient of frequency (TCF) of the device^38^. Therefore, the incident IR power can be readily detected by monitoring the resonance frequency of the device.

As we show in the following, the proposed plasmonic nanomechanical resonant structure provides all the fundamental features necessary for the implementation of uncooled infrared detectors with unprecedented performance (Supplementary Note 2). First, we need to maximize its absorption within the spectrum of interest: to this end, an array of subwavelength patches (Fig. 1b) is patterned within the top metal electrode of the device. Proper patterning of such plasmonic nanostructures in the top metal layer allows the whole device to behave as a spectrally selective and polarization-independent infrared ultrathin absorber^26^, significantly enhancing the electromagnetic field concentration within the AlN dielectric (Supplementary Fig. 3). While a single array of subwavelength patches is fundamentally bound to absorb no more than half of the impinging radiation for symmetry constraints^39^, the presence of the piezoelectric nanoplate allows us to go beyond this limit and absorb a large portion of infrared energy at resonance. At the same time, we need to achieve maximum thermal isolation of the resonant body from the heat sink, which is ensured by minimizing the thickness of the tethers used to support the nanoplate. The anchors of piezoelectric MEMS/NEMS resonators are conventionally composed of a thick and thermally conductive piezoelectric layer, directly patterned on the same layer forming the vibrating body of the resonator^35^, and a thin metal layer employed to route the electrical signal to the actuation electrode integrated in the body of the resonator. On the contrary, here the relatively thick piezoelectric material is completely removed from the anchors, minimizing their thicknesses (ultimately limited by the need of a thin metal layer for electrical routing), resulting in a resonant thermal detector with markedly enhanced responsivity (0.68 Hz nW^−1^). Third, we need to achieve also a low-thermal time constant (440 μs), which is obtained by exploiting the unique properties of high-quality ultrathin AlN films deposited on a Si substrate with a low-temperature sputtering process, enabling low-volume piezoelectric nanoplate resonant structures with a significantly reduced thermal mass. Despite the resonator volume scaling, we are also able to obtain low-noise performance, thanks to the piezoelectric transduction properties of the ultrathin AlN film, which enable efficient on-chip piezoelectric actuation and sensing of a high *Q*>1,000 and high-frequency bulk vibration mode in a free-standing nanoplate, leading to a resonator with very low noise spectral density (∼1.46 Hz Hz^−1/2^ at 100 Hz measurement bandwidth).

The plasmonic metasurface was designed using a transmission line approach, assuming a continuous 100-nm-thick Pt layer beneath the piezoelectric thin film (see Methods). The patch and unit cell dimensions were chosen to provide a Fabry–Perot-like resonance at ∼8.8 μm. While a conventional longitudinal resonance would lead to a significant thickness, severely affecting the mechanical and thermal response of the resonator, in our design we tailored the plasmonic metasurface patterned on top of the grounded AlN nanoplate to have a large capacitive surface reactance *X*_s_=−1/(*ωC*_s_), under a *e*^−*iωt*^ time convention. Stacked on top of a grounded slab, the dominant resonance is achieved when 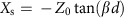, where *Z*_0_ and *β* are the characteristic impedance and propagation constant of the AlN substrate. It confirms that, by tailoring the surface reactance of the plasmonic metasurface to be largely capacitive, it is possible to induce an ultrathin Fabry–Perot resonance in the substrate. It is worth noting that, despite the presence of a small perturbation in the bottom metal layer (interdigitated configuration with two 6-μm-wide gaps rather than a perfectly continuous ground plane), the induced Fabry–Perot-like resonance is mainly determined by the physical dimensions of the gold patches patterned on the top surface of the AlN nanoplate, which are polarization independent^26^.

**Figure 2:**
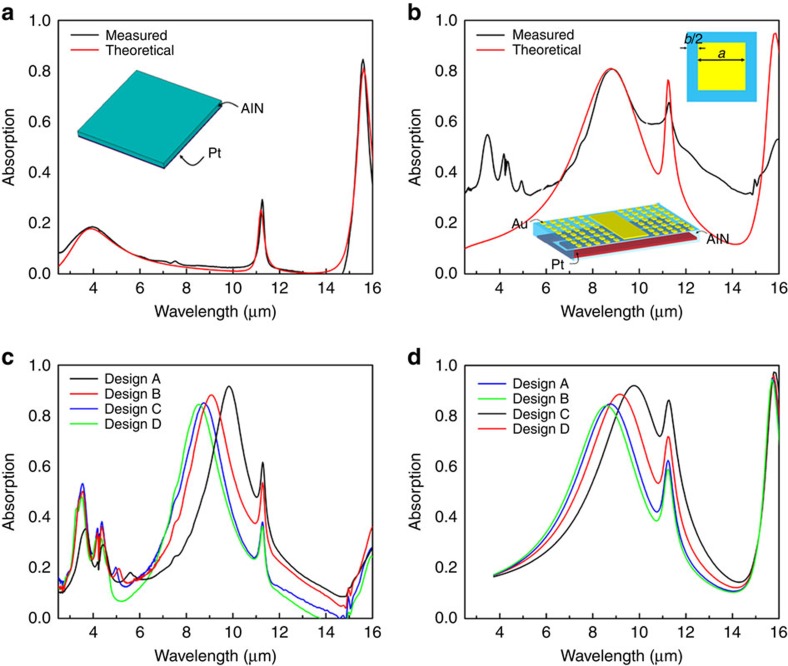
Absorption properties of the proposed plasmonic piezoelectric nanomechanical resonator. (**a**) Simulated (transmission line theory) and measured (FTIR) absorption spectra of a 500-nm-thick AlN slab grounded by a Pt layer (without plasmonic nanostructures). It shows two intrinsic absorption peaks, associated with AlN at 11.3 μm (888 cm^−1^) and 15.5 μm (647 cm^−1^)^40^, and one at 4 μm associated with the resonant structure. (**b**) Simulated and measured absorption spectra of the fabricated plasmonic piezoelectric nanomechanical resonator. The dimensions of the Au patches that compose the metasurface are *a*=1,635 nm, *b*=310 nm, and the thickness of the Au, AlN and Pt layers are 50, 500 and 100 nm, respectively. (**c**,**d**) Measured and simulated absorption properties of the piezoelectric plasmonic resonant structure with varied Au patch sizes, demonstrating its functionality of spectrally elective detection of infrared radiation in the LWIR range. The unit cell sizes are as follows: design A: *a*=1,780 nm, *b*=128 nm; design B: *a*=1,680 nm, *b*=253 nm; design C: *a*=1,640 nm, *b*=313 nm; design D: *a*=1,620 nm, *b*=331 nm.

In our device, the plasmonic nanostructures cover 80% of the top metal layer, as a trade-off between large absorption, achieved by coating the entire layer, and high electromechanical transduction efficiency, achieved by removing a portion of the metasurface and replacing it with continuous metal (Supplementary Note 3). We achieved an electromechanical coupling coefficient, *k*_t_^2^∼1% (Supplementary Fig. 4). Figure 2a,b presents the predicted absorption with and without top subwavelength patches, highlighting how the metasurface can largely increase the absorption at resonance, despite the deeply subwavelength thickness of the device. We also theoretically and experimentally demonstrate that a strong and spectrally selective absorption of long-wavelength infrared (LWIR) radiation, with lithographically determined centre frequency and peak values >85% (over the device area covered by the plasmonic metasurface), can be readily achieved (Fig. 2c,d). Our measurements match very well with the theoretical simulations within the entire band of interest.

### Device fabrication and characterization

On the basis of this design, the proposed infrared detector (Fig. 1) was fabricated using a post-complementary metal-oxide-semiconductor (CMOS) compatible microfabrication process involving a combination of photolithography (four masks) and electron-beam lithography (one step). The Fourier transform infrared (FTIR) absorption spectrum of the device was first measured (see Methods) showing that ∼80% of the impinging optical power (normal incidence to the device surface) is absorbed at the desired spectral wavelength (Fig. 2b), which validates the performance of the designed piezoelectric metasurface. By removing the plasmonic pattern from the device top electrode would lead to negligible absorption, confirming the uniqueness of the proposed design (Fig. 2a).

We also show that, by altering the lateral size of the nanoplasmonic structure (gold patch), it is possible to accurately tailor the absorption peak over a wide range for spectrally selective infrared detection (Fig. 2c,d). We do note the presence of absorption peaks at around 3–4 μm in Fig. 2b,c, which we attribute to an in-plane resonance arising between the fingers of the interdigitated bottom Pt electrode.

**Figure 3:**
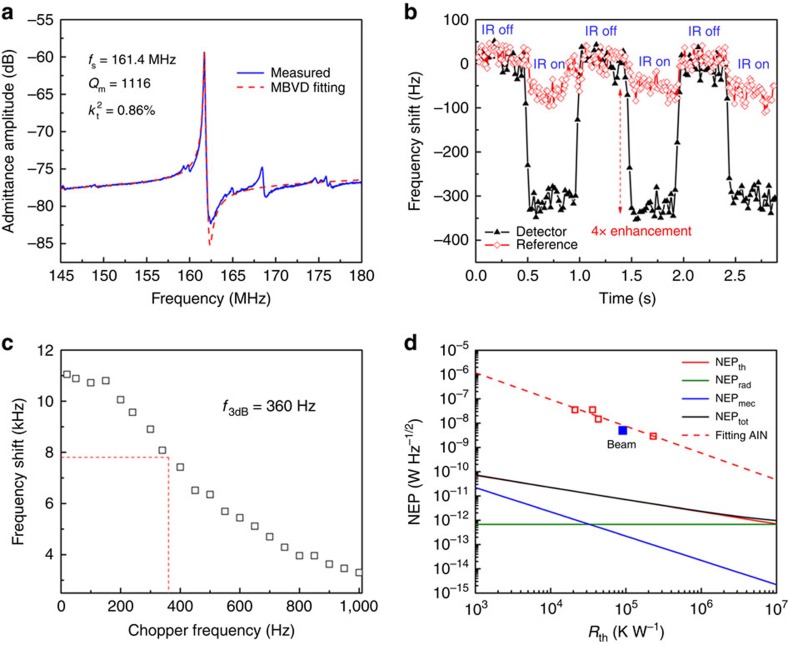
Device performance. (**a**) Measured admittance curve versus frequency and MBVD model fitting of the resonator for *Q*_IR_=0. The extracted values of the MBVD parameters (see Methods) are as follows: *R*_s_=80 Ω; *R*_m_=880 Ω; *L*_m_=1 mH; *C*_m_=1 fF; *R*_0_=2 kΩ; *C*_0_=145 fF. (**b**) Measured response of the plasmonic piezoelectric resonator and a conventional AlN MEMS resonator to a modulated IR radiation emitted by a 1,500-K globar (2–16 μm broadband spectral range). (**c**) Measured frequency response of the detector. The 3dB cutoff frequency, *f*_3 dB_, was found to be 360 Hz, resulting in a time constant *τ*=1/(2*πf*_3 dB_) of 440 μs. (**d**) NEP for different values of thermal resistance (*R*_th_). The solid lines indicate the calculated NEP values associated with each of the three fundamental noise contributions (as expressed in Supplementary Equations 5–6), assuming: resonator area=200 × 75 μm^2^, *ɛ*=1, *T*_0_=300 K, *P*_*c*_=0 dBm, |TCF|=30 p.p.m. K^−1^, *Q*=2,000. The individual data points indicate the measured NEP values of four fabricated AlN resonant plasmonic IR detectors using four different anchor designs (hence four different *R*_th_ values) and a Si_3_N_4_ nanobeam (spectrally selective, static measurement using off-chip optical readout)^34^. IR, infrared.

The electromechanical performance of the resonator was characterized by measuring its admittance versus frequency (Fig. 3a). A high *Q*=1,116 and electromechanical coupling coefficient *k*_t_^2^=0.86% were extracted by equivalent model fitting (Fig. 3a; Methods), demonstrating the unique advantages of the proposed design in terms of high electromechanical transduction efficiency and low loss. The thermal properties (thermal resistance, temperature distribution and TCF) of the infrared detector were characterized by both finite element analysis and experimental verification (Supplementary Figs 5–8; Supplementary Table 1; Supplementary Note 4). The response of the fabricated infrared detector in the LWIR band was characterized using a 1,500-K globar (2–16-μm emission) as an infrared source. For the sake of comparison, the incoming infrared radiation was also detected using a conventional AlN MEMS resonator with same frequency sensitivity to absorbed heat but without plasmonic pattern (hence non-enhanced infrared absorptance). Thanks to its properly engineered optical properties, the piezoelectric plasmonic resonator showed fourfold enhanced responsivity (Fig. 3b), despite its absorption band (full width at half maximum of 1.5 μm) being much narrower than the emission band of the source. With a narrowband source at the frequency of interest, the responsivity would be much larger. The smallest impinging optical power that can be detected was experimentally estimated by measuring the device responsivity, *R*_s_, and noise spectral density (Supplementary Note 5), demonstrating a low noise equivalent power (NEP) ∼2.1 nW Hz^−1/2^ at the designed spectral wavelength (for which infrared absorptance ∼80%). The NEP is arguably considered the most important performance metric for an infrared detector (Supplementary Note 5) and the value measured for the realized proof-of-concept detector proposed here is already comparable to the best commercially available uncooled broadband thermal detectors, while providing unique spectral selectivity in the LWIR band. The response time of the detector was also evaluated by measuring the attenuation of the device response when exposed to infrared radiation modulated at increasingly faster rates (Fig. 3c; Methods), showing a low-thermal time constant, *τ* ∼440 μs.

## Discussion

Differently from more conventional approaches involving the integration of an infrared absorbing material (such as Si_3_N_4_, SiO_2_ or a metal–insulator–metal grating) on top of an optical/mechanical resonant thermal detector^8^, our approach uses an individual plasmonic piezoelectric nanostructure acting simultaneously as absorber, resonator and transducer. It is also worth noting that our proposed plasmonic NEMS resonant sensor exploits a high-frequency on-chip piezoelectric transduction mechanism that has been a key enabler for MEMS technologies. In particular, AlN-based piezoelectric micro-acoustic devices are nowadays the commercial standard used in the radio frequency (RF) front ends of modern smart phones (that is, see Avago Technologies thin-film bulk acoustic resonator (FBAR)). Therefore, the demonstration of a resonant infrared detector based on a bulk-extensional mode plasmonically enhanced piezoelectric resonator, instead of more conventional MEMS, optical resonators or optomechanical structures, is a key advancement over earlier works that really enables the use of these emerging MEMS plasmonic technologies in low-power and miniaturized wireless communication devices. The unique capability of such AlN-based MEMS technology to deliver high-performance and CMOS compatible sensors and RF components makes it the best candidate for the realization of the next-generation miniaturized, low-power, multi-functional and reconfigurable wireless sensing platforms that will be crucial for the development of the internet of things.

Importantly, even though the proposed resonator possesses a unique combination of efficient electromechanical transduction, strong and spectrally selective infrared absorption capability and very low NEP, there is still plenty of room to improve the performance of these piezoelectric plasmonic NEMS devices. First, novel designs could be employed to further improve the absorption of the piezoelectric metasurface to near unity^41^. Second, to reach the thermal fluctuation noise limit (Fig. 3d), all the noise sources contributing to the generation of frequency fluctuations, such as the resonator flicker noise, random walk and drifts^42^, need to be carefully investigated and mitigated. Moreover, the volume of the plasmonic piezoelectric resonator can be further reduced (for instance, by scaling the thickness of the piezoelectric nanoplate, see Supplementary Fig. 9) and its design can be optimized, investigating optimal materials and innovative geometries for the device anchors to increase the thermal resistance up to ∼10^7^ K W^−1^, which is typical of conventional microbolometers. As a result, we expect this technology to achieve NEP in the order of ∼1 pW Hz^−1/2^ (Fig. 3d), thus enabling the implementation of multi-spectral thermal imagers with noise equivalent temperature difference as low as ∼1 mK.

In conclusion, we have demonstrated an uncooled NEMS resonant infrared detector with unique spectrally selective infrared detection capability. Sensing and actuation of a high-frequency (162 MHz) bulk acoustic mode of vibration in a free-standing ultrathin piezoelectric plasmonic metasurface have been demonstrated and exploited for the implementation of a NEMS resonator with unique combined optical and electromechanical properties. By exploiting the piezoelectric properties of AlN thin films, efficient on-chip transduction of the NEMS plasmonic resonant structure has been achieved, eliminating the need for the cumbersome and complex off-chip optical readouts employed in previous devices. Thanks to properly tailored absorption properties, strong and spectrally selective detection of infrared radiation, for an optimized spectral bandwidth centered around 8.8 μm and high thermomechanical coupling between electromagnetic and mechanical resonances in a single ultrathin piezoelectric nanoplate have been experimentally verified. This work sets a milestone towards the development of a new technology platform based on the combination of nanoplasmonics and piezoelectric nanoelectromechanical systems, which can potentially deliver fast (hundreds of μs), high resolution (NEP as low as ∼1 pW Hz^−1/2^) and spectrally selective uncooled infrared detectors suitable for the implementation of high-performance, miniaturized and power efficient infrared/THz spectrometer and multi-spectral imaging systems.

## Methods

### Fabrication

**Figure 4:**
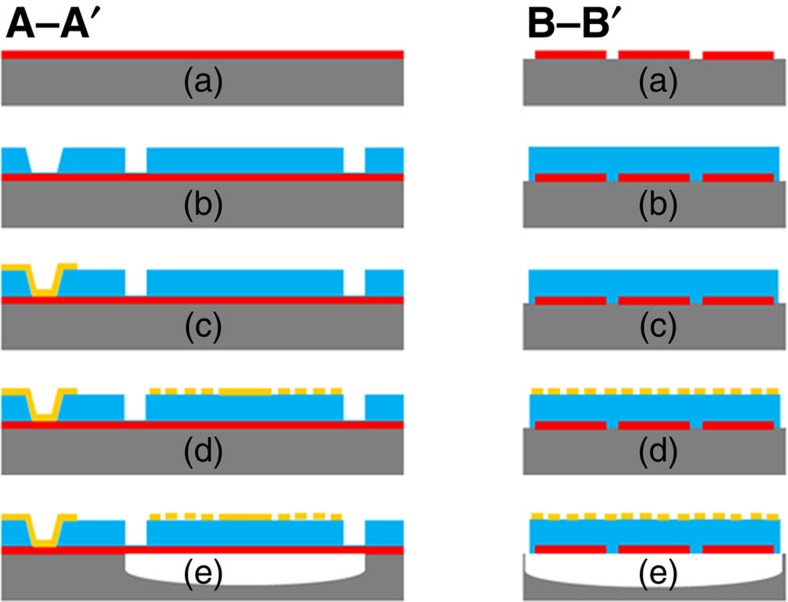
Microfabrication process for the plasmonic piezoelectric NEMS resonant infrared detector. A–A′ and B–B′ denote longitudinal and transversal axis of the device, as illustrated in Fig. 1.

The plasmonic piezoelectric resonant infrared detector was fabricated using a post-CMOS compatible microfabrication process involving a combination of photolithography (four masks) and electron-beam lithography (one step), as illustrated in Fig. 4. The fabrication started with a high-resistivity (resistivity >20,000 Ω m) 4-inch silicon wafer: (a) 100-nm-thick Pt was sputter-deposited and patterned by lift-off process to define the bottom IDT; (b) 500-nm-thick high-quality *c* axis orientated AlN film was sputter-deposited on top of the Pt IDT, and wet etched in H_3_PO_4_ to open the vias to get access to the bottom electrode and dry etched by inductively coupled plasma in Cl_2_-based chemistry to define the shape of the AlN resonator; (c) 100-nm-thick Au was deposited by electron-beam deposition and patterned by lift-off process to define the probing pad; (d) 50-nm-thick Au was deposited by electron-beam deposition and patterned by electron-beam lithography and lift-off process to define the nanoplasmonic metasurface; (e) the Si substrate underneath the resonator was etched by xenon difluoride (XeF_2_) to completely release the device.

### Modelling

#### Nanoplasmonic piezoelectric resonant infrared detector—electrothermal equivalent circuit

**Figure 5:**
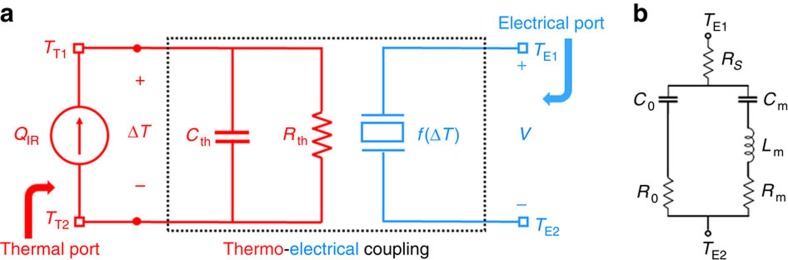
Electrothermal equivalent circuit of the device. (**a**) Equivalent thermoelectrical circuit of the nanoplasmonic piezoelectric NEMS resonator. (**b**) Modified Butterworth–Van Dyke (MBVD) equivalent circuit.

The proposed plasmonic piezoelectric NEMS resonant infrared detector is modelled by a two-port network with both electrical (voltage, *V*, used to drive the electromechanical resonance) and thermal inputs (infrared power, *Q*_IR_, absorbed in the piezoelectric resonant structure), as shown in Fig. 5a. At the thermal port, the free-standing resonant structure is simply modelled as a thermal mass, with thermal capacitance *C*_th_, coupled with the heat sink at a constant temperature *T*_0_ via the thermal conductance *G*_th_ (thermal resistance, *R*_th_=1/*G*_th_). The thermal capacitance is intrinsically related to the properties and overall volume of the material stack forming the vibrating structure (*C*_th_*=c*·*v*·*ρ*; where *c* is the specific heat capacity, *v* is the volume and *ρ* is the material density). The thermal conductance is instead mainly determined by the geometry and material properties of the tethers connecting the free-standing vibrating body of the device to the substrate (Supplementary Note 2). At the electrical port, the resonator is modelled with a modified Butterworth–Van Dyke (MBVD) equivalent circuit model^43^, shown in Figure 5b. The MBVD circuit consists of two branches in parallel: an acoustic branch composed by the series combination of the motional resistance, *R*_m_ (quantifying dissipative losses), motional capacitance, *C*_m_ (inversely proportional to the stiffness) and motional inductance, *L*_m_ (proportional to the mass); and an electrical branch composed by the series combination of the capacitance, *C*_0_ (capacitance between the device terminals), and resistance, *R*_0_ (representing the dielectric loss). A series resistance, *R*_s_, is also included in the circuit to represent the electrical loss associated with the metal electrodes and routing (Fig. 5b).

#### Nanoplasmonic piezoelectric resonant infrared detector—electromagnetic model

This section briefly presents the analysis and design of the proposed plasmonic nanomechanical resonator from an electromagnetic point of view. Specifically, this analysis permits obtaining the frequency-dependent absorption coefficient of the resonator, *η*, which determines the resonator increase of temperature (Δ*T)* when infrared radiation is impinging onto the detector (Supplementary Note 2).

**Figure 6:**
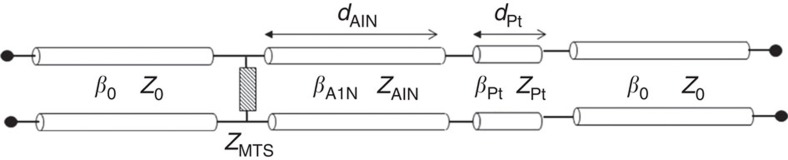
Electromagnetic circuit model of the piezoelectric resonator. A normally incident Transverse electromagnetic (TEM) wave impinges on the structure shown in Fig. 1. The outer transmission line sections represent the free space, *Z*_MTS_, is the surface impedance of the array of gold patches (metasurface), and the inner transmission line section takes into account the AlN dielectric and the ground platinum layer, respectively. Each section is characterized by its characteristic impedance *Z* and propagation constant *β*.

Thanks to the structure symmetry and assuming a continuous Pt layer beneath the AlN, the analysis of a single unit cell of the metasurface suffices to investigate the electromagnetic behaviour of the whole device. This analysis is carried out using the equivalent transmission line of Fig. 6. This circuit is totally rigorous^44^,^45^, that is, exactly equivalent to solving Maxwell's equations, assuming that (i) a transverse electromagnetic (TEM) wave is normally impinging on the resonator and (ii) the operation frequency is well below the cutoff frequency of the higher-order modes excited in the metasurface. Note that these two conditions are indeed fulfilled in this case.

The characteristic impedance and propagation constant of the different transmission line sections involved in the model are


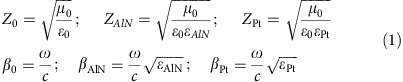


where the subscripts ‘0', ‘AlN' and ‘Pt' denote free space, aluminum nitrate dielectric and Pt, respectively, ‘*c*' is the speed of light, 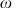 is the radial frequency and 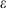 is permittivity, respectively. In addition, the Pt layer is modelled following a simple Drude model


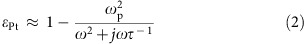


with plasma frequency *ω*_p_=7.88 × 10^15^ rad s^−1^ and relaxation time *τ*=7.8 fs (ref. 46). We characterize the aluminum nitrate substrate using a Lorentzian permittivity model as


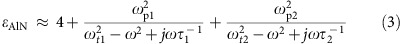


with *ω*_p1_=8.663 × 10^13^ rad s^−1^, *ω*_*t*1_=1.6894 × 10^14^ rad s^−1^, *τ*_1_=0.2367, ps, *ω*_p2_=2.5115 × 10^14^ rad s^−1^, *ω*_*t*2_=1.2558 × 10^14^ rad s^−1^, *τ*_2_=0.3185, ps. The electromagnetic model of the array of gold patches is very challenging and there are no closed-form expressions available for a general case. However, in case of electrically small unit cells (that is, *a*+*b*<<*λ*_0_, being *λ*_0_ the operating wavelength) and densely packed planar patches (b<<*a*), the surface impedance of the array of gold patches can be approximated by^44^


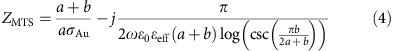


where *σ*_Au_ is the gold conductivity and *ɛ*_eff_=(1+*ɛ*_AlN_)/2 is the effective permittivity surrounding the metasurface. We have numerically verified that equation 4 provides accurate results in the specific metasurfaces under consideration. However, note that the near field created by the actual thickness of the gold patches (∼50 nm) slightly decreases the effective distance between the patches. This phenomenon, common in microwave and optics^47^, is easily taken into account using an effective separation distance *b* slightly lower than the physical one.

The proposed circuit is analysed using an analytical transfer-matrix approach, thus providing the absorption coefficient of the resonator versus frequency. The metasurface employed to cover the AlN resonator is designed in two steps: (i) the input impedance and absorption of the AlN slab grounded by the Pt layer are computed using the equivalent circuit of Fig. 6 in the absence of the metasurface (that is, *Z*_MTS_≈∞), and (ii) the dimensions of the unit cell and patches of the metasurfaces are chosen with the help of (equations 2, 3, 4) aiming to match the input impedance of the structure to the free-space impedance at the operation frequency, therefore maximizing the absorption coefficient. Finally, the electromagnetic behaviour of the designed structure is confirmed using the full-wave commercial software Computer Simulation Technology (CST) mimcrowave studio.

It is important to point out the good agreement between theory and measurement (Fig. 2a,b), especially taking into account that the fabricated device does not have a continuous Pt layer, but a patterned one (patterned IDT with a partial coverage of ∼70%, which is needed to excite a 160-MHz contour-extensional mode of vibration in the plasmonic piezoelectric nanoplate). However, the influence of this pattern is limited because (i) most of the unit cells (around 70%) are grounded by a continuous Pt layer and (ii) it basically introduces a small capacitance to the ultrathin Pt layer electromagnetic response. We have further verified with CST full-wave simulations that the influence of this pattern is negligible.

### Measurements

The electromechanical performance of the plasmonic piezoelectric resonator was characterized by measuring its equivalent electrical admittance with an Agilent E5071C vector network analyzer after performing an open-short-load calibration on a standard substrate. The scattering parameter, *S*_11_, of this one-port electrical network was first measured and then converted into the admittance, *Y*, according to: *Y*=(1/50)(1+*S*_11_)/(1−*S*_11_). The values of the equivalent circuit elements were extracted from the measurement by MBVD model fitting. The mechanical quality factor, *Q* (inversely proportional to the mechanical damping affecting the resonator), and electromechanical coupling coefficient, *k*_t_^2^ (a numerical measure of the conversion efficiency between electrical and mechanical energy in an electromechanical resonator), were extracted according to: *Q*=(1/*R*_m_)(*L*_m_/*C*_m_)^0.5^, and *k*_t_^2^=(*π*^*2*^/8)(*C*_m_/*C*_0_), respectively^48^. The device admittance amplitude–frequency nonlinearity was measured by monitoring the admittance amplitude versus frequency shift for different levels of RF power. Further details about the measurement and analysis can be found in the Supplementary Note 5.

The reflectance, *R*, spectra of the fabricated nanoplasmonic metasurface and plasmonic piezoelectric resonator were measured using a Bruker V70 Fourier transform infrared coupled with a Hyperion 1000 microscope. A reflective gold mirror was used as a background for calibration. The absorption spectrum, *A*, of each sample was then directly obtained as 1−*R*, assuming minimum transmission through the sample, which is a reasonable assumption given the large metal coverage of both the top Au plasmonic structures and bottom Pt electrode.

The response of the fabricated infrared detector in the LWIR band (Fig. 3b) was characterized using a 1,500-K globar (2–16-μm emission) as an infrared source coupled with a Bruker Hyperion 1000 microscope for focusing the infrared beam onto the device. The incident infrared radiation was modulated by an optical chopper at 1 Hz. Measurement bandwidth (intermediate frequency (IF) bandwidth) of the network analyzer was set to 100 Hz to obtain the best noise performance. All experiments were performed in an open environment in ambient temperature and pressure.

The thermal time constant of the detector was evaluated by measuring the attenuation of the device response when exposed to infrared radiation modulated at increasingly faster rates (Fig. 3c). An EOS Photonics ∼5-μm continuous-wave quantum cascade laser with a relatively high output power (∼50 mW) was employed as a heat source. The infrared radiation emitted by the quantum cascade laser was modulated using an optical chopper (modulation frequency was varied from 1 Hz to 1 kHz) and focused onto the infrared detector using a zinc selenide (ZnSe) lens (with 70% transmission for infrared radiation in the 0.6–16 μm spectral range). The 3dB cutoff frequency, *f*_3 dB_, was found to be 360 Hz, resulting in a time constant *τ*=1/(2π*f*_3 dB_) of 440 μs (Fig. 3c).

**Figure 7:**
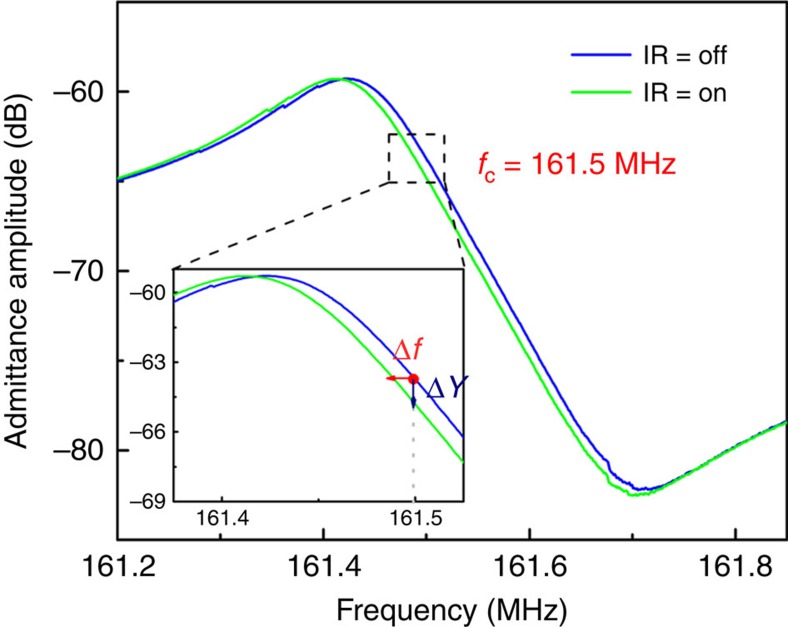
Measured admittance curves versus frequency of the resonator for IR on and off. The inset shows the zoomed in admittance shift around the resonance frequency. The exciting frequency of 161.5 MHz is also marked at which the frequency response of the device was measured. IR, infrared.

The transient responses of the device were measured by exciting the resonator at a single frequency, *f*_c_=161.5 MHz, for which the slope of admittance amplitude curve versus frequency is maximum (121.3 dB MHz^−1^)^7^, and by monitoring the variations over time of the device admittance amplitude (Δ*Y*) using our network analyzer. The admittance amplitude change was then converted to frequency change (Δ*f*) by multiplying the slope of the admittance versus frequency (Fig. 7). The measurement bandwidth (IF bandwidth) of the network analyzer was set to 10 kHz (sampling time of 0.1 ms), which is small enough compared with the thermal time constant of the detector.

## Additional information

**How to cite this article:** Hui, Y. *et al*. Plasmonic piezoelectric nanomechanical resonator for spectrally selective infrared sensing. *Nat. Commun.* 7:11249 doi: 10.1038/ncomms11249 (2016).

## Supplementary Material

Supplementary Information Supplementary Figures 1-9, Supplementary Table 1, Supplementary Notes 1-5 and Supplementary References.

## References

[b1] Reine M. B. HgCdTe photodiodes for IR detector: a review. Proc. SPIE 4288, 266–277 (2001).

[b2] Kozlowski Lester J. . Recent advances in staring hybrid focal plane arrays: comparison of HgCdTe, InGaAs, and GaAs/AlGaAs detector technologies. Proc. SPIE 2274, 93–116 (1994).

[b3] Rogalski A. Infrared detectors: status and trends. Prog. Quant. Electron. 27, 59–210 (2003).

[b4] Chen C., Yi X., Zhao X. & Xiong B. Characterization of VO_2_ based uncooled microbolometer linear array. Sens. Actuators A 90, 212–214 (2001).

[b5] Schaufelbuhl A. . Uncooled low-cost thermal imager based on micromachined CMOS integrated sensor array. J. Microelectromech. Syst. 10, 503–510 (2001).

[b6] Kang D. H., Kim K. W., Lee S. Y., Kim Y. H. & Keun Gil S. Influencing factors on the pyroelectric properties of Pb (Zr, Ti) O_3_ thin film for uncooled infrared detector. Mater. Chem. Phys. 90, 411–416 (2005).

[b7] Hui Y. & Rinaldi M. Fast and high resolution thermal detector based on an aluminum nitride piezoelectric microelectromechanical resonator with an integrated suspended heat absorbing element. Appl. Phys. Lett. 102, 093501 (2013).

[b8] Watts M. R., Shaw M. J. & Nielson G. N. Optical resonators: Microphotonic thermal imaging. Nat. Photon. 1, 632–634 (2007).

[b9] Masmanidis S. C. . Multifunctional nanomechanical systems via tunably coupled piezoelectric actuation. Science 317, 780–783 (2007). 17690289 10.1126/science.1144793

[b10] Li M., Tang H. X. & Roukes M. L. Ultra-sensitive NEMS-based cantilevers for sensing, scanned probe and very high-frequency applications. Nat. Nanotechnol. 2, 114–120 (2007). 18654230 10.1038/nnano.2006.208

[b11] Niklaus F., Vieider C. & Jakobsen H. in Photonics Asia 2007 68360D (International Society for Optics and Photonics, 2007).

[b12] Graf A., Arndt M., Sauer M. & Gerlach G. Review of micromachined thermopiles for infrared detection. Meas. Sci. Technol. 18, R59 (2007).

[b13] Exner A. T., Pavlichenko I., Lotsch B. V., Scarpa G. & Lugli P. Low-cost thermo-optic imaging sensors: a detection principle based on tunable one-dimensional photonic crystals. ACS Appl. Mater. Interfaces 5, 1575–1582 (2013). 23286349 10.1021/am301964y

[b14] Tallur S. & Bhave S. A. A silicon electromechanical photodetector. Nano Lett. 13, 2760–2765 (2013). 23706144 10.1021/nl400980u

[b15] Hui Y. & Rinaldi M. in *The 17th International Conference on Solid-State Sensors, Actuators and Microsystems* (TRANSDUCERS & EUROSENSORS XXVII), 2013 Transducers & Eurosensors XXVII: 968–971 (Barcelona, Spain, 2013).

[b16] Qian Z., Hui Y., Liu F., Kai S. & Rinaldi M. in *18th International Conference on Solid-State Sensors, Actuators and Microsystems* (TRANSDUCERS), 2015 Transducers 1429–1432 (Anchorage, AK, USA, 2015).

[b17] Gokhale V. J., Myers P. D. & Rais-Zadeh M. in *IEEE Sensors*, 982–985 (Valencia, Spain, 2014).

[b18] Kildishev A. V., Boltasseva A. & Shalaev V. M. Planar photonics with metasurfaces. Science 339, 1232009 (2013). 23493714 10.1126/science.1232009

[b19] Yu N. & Capasso F. Flat optics with designer metasurfaces. Nat. Mater. 13, 139–150 (2014). 24452357 10.1038/nmat3839

[b20] Zhao Y., Belkin M. & Alù A. Twisted optical metamaterials for planarized ultrathin broadband circular polarizers. Nat. Commun. 3, 870 (2012). 22643897 10.1038/ncomms1877

[b21] Pfeiffer C. & Grbic A. Metamaterial Huygens' surfaces: tailoring wave fronts with reflectionless sheets. Phys. Rev. Lett. 110, 197401 (2013). 23705738 10.1103/PhysRevLett.110.197401

[b22] Esquius-Morote M., Gomez-Diaz J. S. & Perruisseau-Carrier J. Sinusoidally modulated graphene leaky-wave antenna for electronic beamscanning at THz. IEEE Trans. THz Sci. Technol. 4, 116–122 (2014).

[b23] Pozar D. Flat lens antenna concept using aperture coupled microstrip patches. Electron. Lett. 32, 2109–2111 (1996).

[b24] Monticone F., Estakhri N. M. & Alù A. Full control of nanoscale optical transmission with a composite metascreen. Phys. Rev. Lett. 110, 203903 (2013). 25167411 10.1103/PhysRevLett.110.203903

[b25] Yu N. . Light propagation with phase discontinuities: generalized laws of reflection and refraction. Science 334, 333–337 (2011). 21885733 10.1126/science.1210713

[b26] Mosallaei H. & Sarabandi K. in *Antennas and Propagation Society International Symposium, 2005 IEEE*, 615–618 (Washington, DC, USA, 2005).

[b27] Argyropoulos C., Le K. Q., Mattiucci N., D'Aguanno G. & Alu A. Broadband absorbers and selective emitters based on plasmonic Brewster metasurfaces. Phys. Rev. B 87, 205112 (2013).

[b28] Ou J.-Y., Plum E., Zhang J. & Zheludev N. I. An electromechanically reconfigurable plasmonic metamaterial operating in the near-infrared. Nat. Nanotechnol. 8, 252–255 (2013). 23503091 10.1038/nnano.2013.25

[b29] Yamaguchi K., Fujii M., Okamoto T. & Haraguchi M. Electrically driven plasmon chip: active plasmon filter. Appl. Phys. Express 7, 012201 (2014).

[b30] Dennis B. . Compact nanomechanical plasmonic phase modulators. Nat. Photon. 9, 267–273 (2015).

[b31] Valente J., Ou J.-Y., Plum E., Youngs I. J. & Zheludev N. I. A magneto-electro-optical effect in a plasmonic nanowire material. Nat. Commun. 6, (2015). 10.1038/ncomms8021 PMC4421854 25906761

[b32] Ogawa S., Okada K., Fukushima N. & Kimata M. Wavelength selective uncooled infrared sensor by plasmonics. Appl. Phys. Lett. 100, 021111 (2012).

[b33] Talghader J. J., Gawarikar A. S. & Shea R. P. Spectral selectivity in infrared thermal detection. Light Sci. Appl. 1, e24 (2012).

[b34] Yi F., Zhu H., Reed J. C. & Cubukcu E. Plasmonically enhanced thermomechanical detection of infrared radiation. Nano Lett. 13, 1638–1643 (2013). 23484543 10.1021/nl400087b

[b35] Rinaldi M. & Piazza G. in *2011 Joint Conference of the IEEE International on Frequency Control and the European Frequency and Time Forum (FCS)*, 1–5 (San Fransisco, CA, USA, 2011).

[b36] Hui Y., Qian Z., Hummel G. & Rinaldi M. in *Proceedings of the 2014 Solid-State Sensors, Actuators and Microsystems Workshop (Hilton Head 2014)* 387–391 (Hilton Head Island, SC, USA, 2014) 387–390 (2014).

[b37] Hui Y. & Rinaldi M. in *28th IEEE International Conference on Micro Electro Mechanical Systems (MEMS)* 984–987 (Estoril, Portugal, 2015).

[b38] Kuypers J. H., Lin C.-M., Vigevani G. & Pisano A. P. in *2008 IEEE International Frequency Control Symposium*, 240–249 (Honolulu, HI, USA, 2008).

[b39] Thongrattanasiri S., Koppens F. H. & de Abajo F. J. G Complete optical absorption in periodically patterned graphene. Phys. Rev. Lett. 108, 047401 (2012). 22400887 10.1103/PhysRevLett.108.047401

[b40] Ibáñez J. . Far-infrared transmission in GaN, AlN, and AlGaN thin films grown by molecular beam epitaxy. J. Appl. Phys. 104, 033544 (2008).

[b41] Hendrickson J., Guo J., Zhang B., Buchwald W. & Soref R. Wideband perfect light absorber at midwave infrared using multiplexed metal structures. Optics Lett. 37, 371–373 (2012). 10.1364/OL.37.000371 22297356

[b42] Rubiola E. Phase Noise and Freqeuncy Stability in Oscillators Cambridge Univ. Press (2008).

[b43] Larson J. D. III, Bradley R., Wartenberg S. & Ruby R. C. in *IEEE Ultrasonics Symposium*, 863–868 (San Juan, 2000).

[b44] Tretyakov S. Analytical Modeling in Applied Electromagnetics Artech House (2003).

[b45] Padooru Y. R., Yakovlev A. B., Kaipa C. S., Medina F. & Mesa F. Circuit modeling of multiband high-impedance surface absorbers in the microwave regime. Phys. Rev. B 84, 035108 (2011).

[b46] Rakic A. D., Djurišic A. B., Elazar J. M. & Majewski M. L. Optical properties of metallic films for vertical-cavity optoelectronic devices. Appl. Opt. 37, 5271–5283 (1998). 18286006 10.1364/ao.37.005271

[b47] Pozar D. M. Microwave Engineering John Wiley & Sons (2009).

[b48] Rinaldi M., Zuniga C., Zuo C. & Piazza G. in *IEEE Transactions on Ultrasonics, Ferroelectrics and Frequency Control*, Vol. 57, 38–45 (2010). 10.1109/TUFFC.2010.1376 20040424

