# Supplementary material for: Plasmonic piezoelectric nanomechanical resonator for spectrally selective infrared sensing
Source: Nat Commun. 2016 Apr 15;7:11249. doi: 10.1038/ncomms11249 (PMC4835539; doi:10.1038/ncomms11249)
Supplement: Supplementary Information — Supplementary Figures 1-9, Supplementary Table 1, Supplementary Notes 1-5 and Supplementary References. [file ncomms11249-s1.pdf]

Supplementary Figures

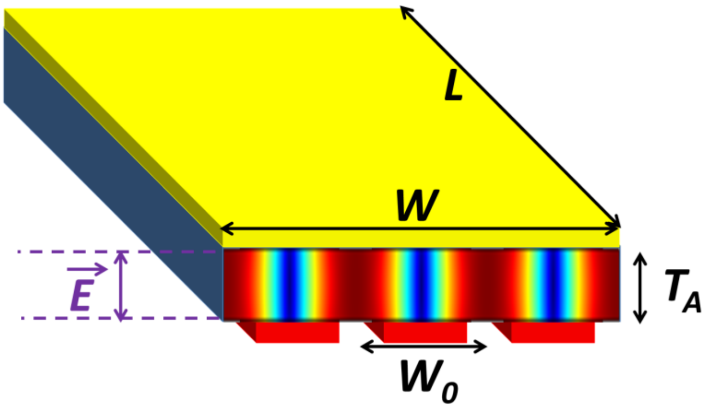

Supplementary Figure 1 | 3D representation of an AlN NPR with bottom inter-digital transducers and top floating metal plate. The 2D FEM simulated lateral-extensional mode of vibration (total displacement) is superimposed to the AlN film.

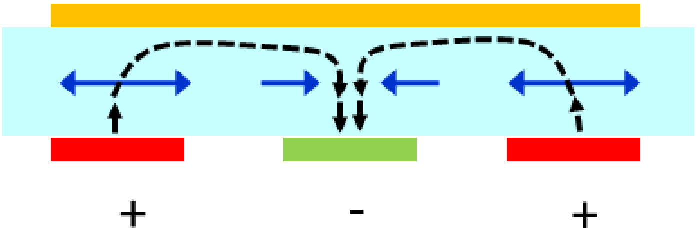

Supplementary Figure 2 | Schematic illustration of the Lateral Field Excitation (LFE) scheme of the AlN NPR. Black dash lines represent the electric field and blue arrows represent the strain induced by the d_{31} piezoelectric coefficient.

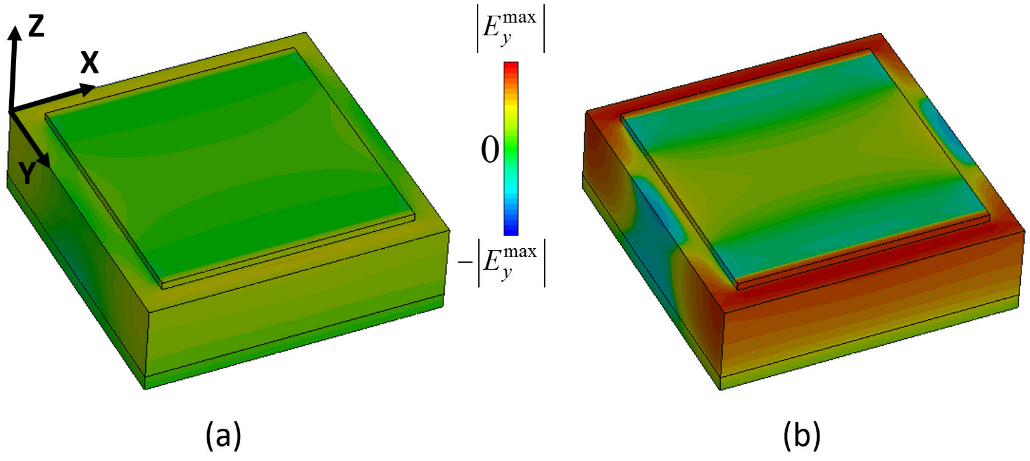

Supplementary Figure 3 | Simulated electric field distribution. Electric field distribution (y-component, logarithm scale) within one unit cell of the proposed nanomechanical resonator (see Fig. 1 of the main manuscript) at (**a**) 5 μm and (**b**) 8.8 μm. The structure is illuminated from the top assuming a y-polarized plane wave.

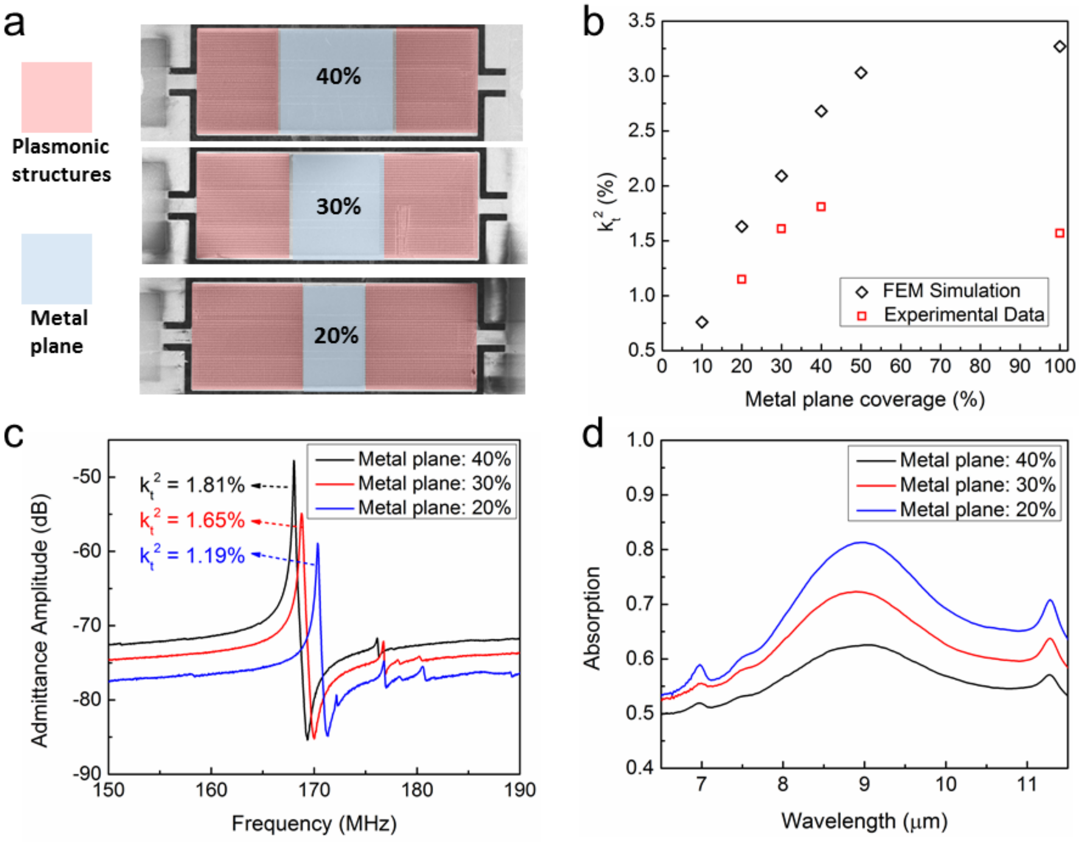

Supplementary Figure 4 | Optimization of device design. (**a**) SEM image of nanoplasmonic piezoelectric resonators (supported by conventional AlN/Pt anchors) with different plasmonic nanostructures and metal planes coverage; (**b**) 3D finite element method (FEM) simulation and experimental verification of the electromechanical coupling coefficient (k_t^2) dependence on the metal plane coverage; (**c**) Electrical performance of the resonators with different metal plane coverage; (**d**) FTIR measured absorption of the resonators with different metal plane coverage.

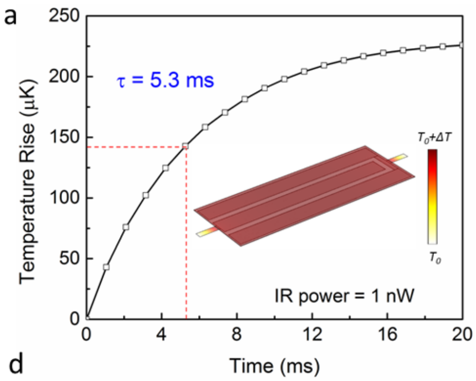

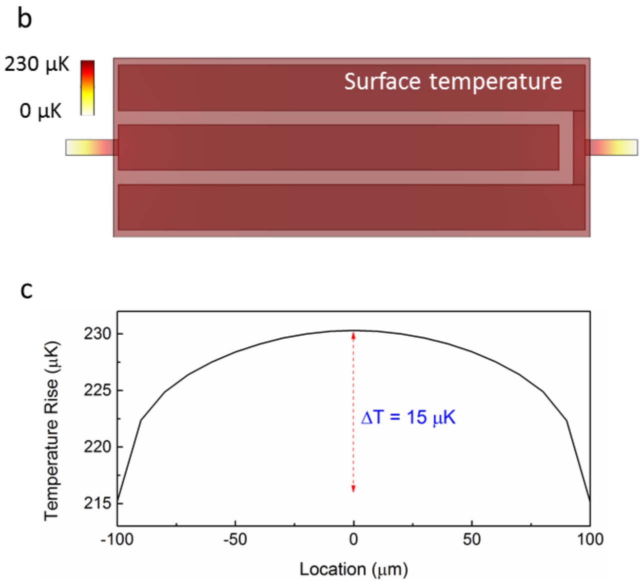

d

Material	k ($Wm^{-1}K^{-1}$)	c ($Jkg^{-1}K^{-1}$)	ρ (kgm^{-3})	C_{th} (nJK^{-1})
AlN	80	740	3260	18.09
Pt	71.6	133	21450	3.17
Au	310	129	19300	2.19
Air	0.024	1010	1.2	1.8

Supplementary Figure 5 | Thermal characteristics (**a**) 3D FEM simulated transient response of the IR detector to an input power of 1 nW, giving a thermal time constant of 5.3 ms; (**b**)-(**c**) Simulated temperature profile cross the resonator for 1 nW IR power, showing a temperature variation of only 15 µK; (**d**) Thermal properties of the materials used for the calculation and simulation.

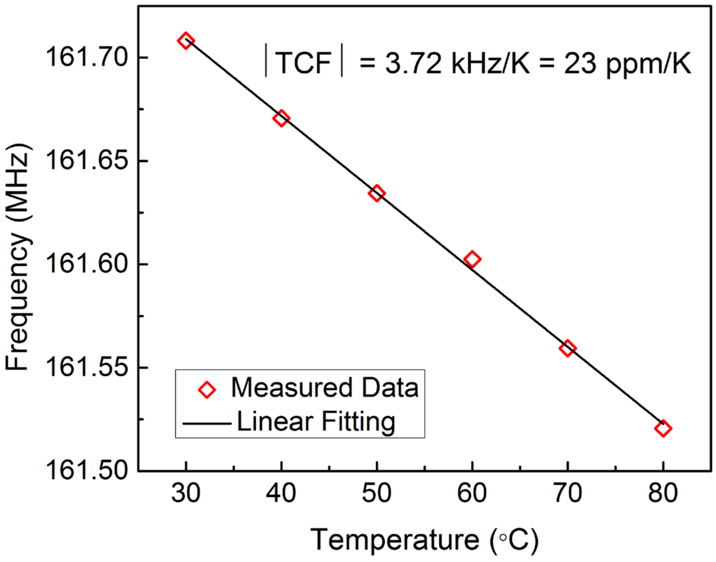

Supplementary Figure 6 | Measured temperature coefficient of frequency of the fabricated plasmonic piezoelectric NEMS resonator.

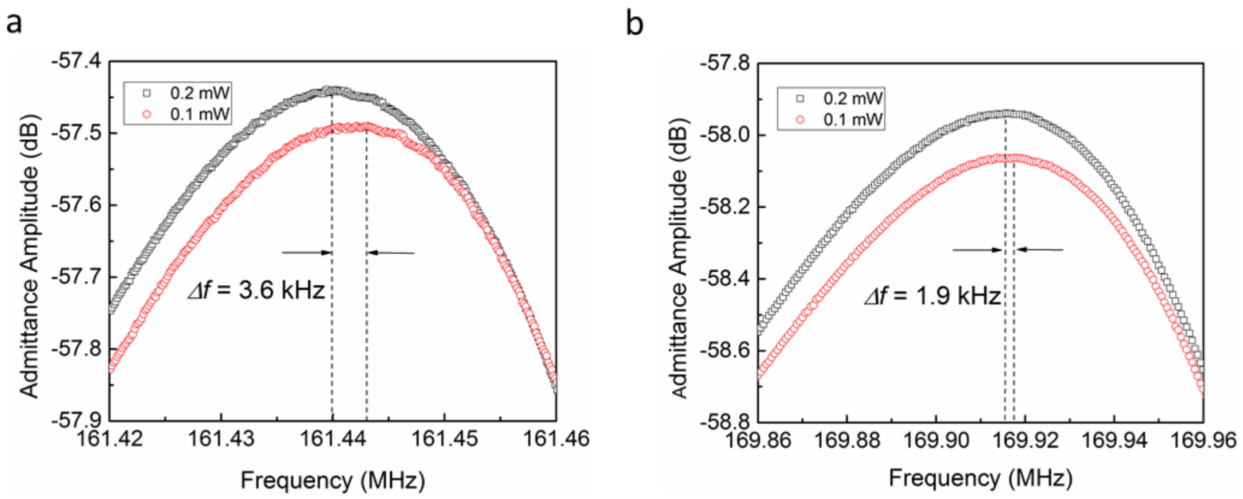

Supplementary Figure 7 | Frequency response to different input RF power. Measured frequency response of (**a**) the IR detector with Pt anchors and (**b**) a reference resonator with conventional AlN/Pt anchors for different levels of input RF power.

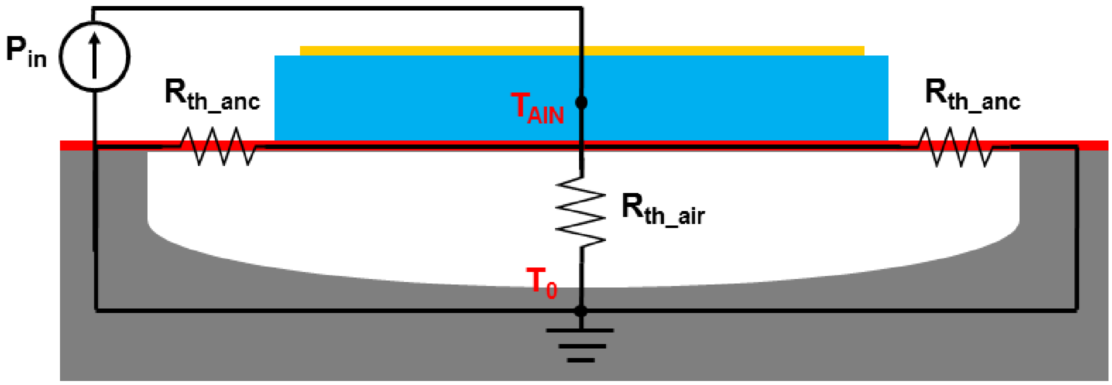

Supplementary Figure 8 | Equivalent thermal circuit of the IR detector with Pt anchors. P_{in} is the input IR power, T_{AlN} and T_0 are the temperature of the AlN resonator and heat sink (Si substrate), respectively. R_{th_anc} and R_{th_air} are the thermal resistance of the Pt anchors and air, respectively.

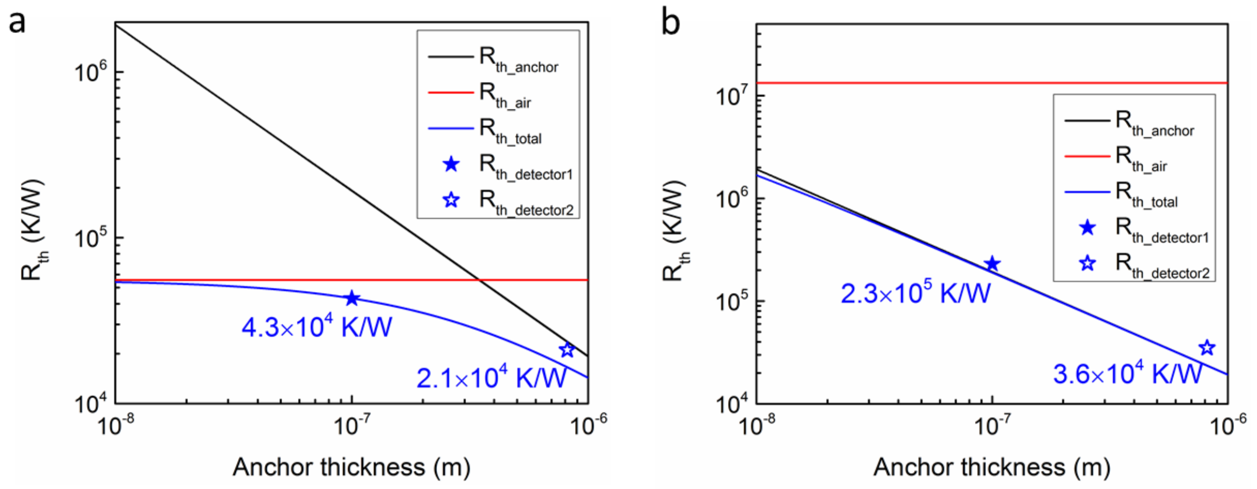

Supplementary Figure 9 | Thermal resistance. Thermal resistance associated to the anchors ($R_{\mathrm{th\_anchor}}$) and the air gap ($R_{\mathrm{th\_air}}$) and total thermal resistance ($R_{\mathrm{th\_tot}}$, parallel combination of $R_{\mathrm{th\_anchor}}$ and $R_{\mathrm{th\_air}}$) for different thicknesses of the device anchors in air (**a**) and vacuum (1 mTorr) (**b**). The measured thermal resistances of Detector1 (with Pt anchors) and Detector2 (with conventional AlN/Pt anchors) in air (**a**) and FEM simulated in vacuum (**b**) are marked in the respective graphs.

Supplementary Table

Supplementary Table 1. Measured parameter values used for the extraction of thermal resistance.

	$\Delta P_c (mW)$	Δf *(kHz)*	*Z (Ω)*	*Γ*	*TCF (ppm/K)*	$f_0 (MHz)$	R_{th} *(K/W)*
Detector1	0.1	3.6	785	0.880	23	161.4	4.3×10^4
Detector2	0.1	1.9	745	0.875	23	169.9	2.1×10^4

Supplementary Notes

Supplementary Note 1. AlN nano-plate resonator

The core of the proposed nanoplasmonic piezoelectric IR detector is an aluminum nitride (AlN) nano-plate resonator (NPR). Supplementary Fig. 1 illustrates a 3-dimensional (3D) representation of a conventional AlN NPR using a lateral field excitation (LFE) scheme (Supplementary Fig. 2). A high quality, c-axis orientated AlN thin film (500 nm thick in this work) is sandwiched between the bottom interdigitated transducer (IDT) and top electrically floating metal plate. The proposed nanoplasmonic piezoelectric NEMS resonator can be modeled in the electrical domain with a modified Butterworth Van Dyke (MBVD) equivalent circuit model[1], shown in Fig. 5b of the main text. The series resonance occurs at the frequency f_s, for which the impedance of the motional capacitance and motional inductance cancel with each other, resulting in a minimum impedance (maximum admittance), while the parallel resonance occurs at the frequency f_p, for which the impedance of the circuit is maximum (minimum admittance).

The two most important parameters to evaluate the performance of an AlN NPR are the quality factor, Q, and electromechanical coupling coefficient, k_t^2. The quality factor is a dimensionless parameter representing the ratio of the energy stored in the vibrating resonant structure to the energy dissipated per cycle by the damping processes (the higher is the Q, the lower is the energy loss), while the electromechanical coupling coefficient is a numerical measure of the conversion efficiency between the electrical and mechanical energy in the electromechanical resonator. The typical values of Q and k_t^2 for ultrathin (≤ 500 nm) AlN NPRs employing LFE scheme (Supplementary Fig. 2) are around 1000 ~ 2000 and 1% ~ 2%, respectively[2]. For a given geometrical capacitance, C_0, and operating frequency, ω_0, the motional resistance (R_m), capacitance (C_m) and inductance(L_m) of the resonator are determined by the values of Q and k_t^2 as

$$R_m = \frac{1}{\omega_0 C_0 k_t^2 Q} \qquad (1)$$

$$C_m = \frac{8}{\pi^2} k_t^2 C_0 \qquad (2)$$

$$L_m = \frac{1}{\omega_0^2 C_m} \qquad (3)$$

In particular the device equivalent motional resistance, R_m, is inversely proportional to the $k_t^2 \cdot Q$ product (Supplementary Eq. 1). The achievement of a low value of motional resistance, in a radio frequency electromechanical resonator, is of crucial importance for the direct interface of the device with compact and low-power 50-Ω electronics[3]. Therefore, the figure of merit (*FOM*) of an electromechanical resonator is defined as the $k_t^2 \cdot Q$ product.

It is worth to note that the fabrication process of an AlN nano-plate resonator is post-CMOS compatible. "post-CMOS compatible process" means that the micro-fabrication process starts with a CMOS wafer and only uses materials which are post CMOS compatible (can be deposited with temperature < 400 °C and dry etched using standard CMOS tools). Previous paper[4] has shown that the current AlN resonant technology employed in this work is post-CMOS compatible. Note that the platinum bottom electrode and gold structures can easily be replaced with standard CMOS metals, such as titanium, titanium nitride, and tungsten.

Supplementary Note 2. Design considerations

The proposed plasmonic piezoelectric NEMS resonant IR detector is modeled with a two-port network with both electrical and mechanical inputs, as shown in Fig. 5a in the main text. When the IR radiation is absorbed by the plasmonically enhanced resonant nano-plate, the temperature of the device increases according to

$$\Delta T = \frac{\eta Q_p}{\sqrt{{G_{th}}^2 + \omega^2 {C_{th}}^2}} \qquad (4)$$

where Q_p is the incident IR power, η is the absorption coefficient of the plasmonic resonant nano-plate, ω is the modulation frequency of the incident IR radiation, G_th and C_th are the thermal conductance and thermal capacitance of the device, respectively. Such IR induced temperature rise results in a shift, Δf, in the mechanical resonance frequency of the structure (Fig. 1a in the main text), given by

$$\Delta f = f_0 \cdot TCF \cdot \Delta T \qquad (5)$$

which arises due to the intrinsically large temperature coefficient of frequency of the piezoelectric resonant nano-plate (the TCF of AlN resonators is typically in the order of -30 ppm·K^{-1} for AlN plates thinner than 1 μm). Using these expressions, the detector overall responsivity for slowly modulated impinging IR radiation can be expressed as

$$R_s = \frac{\Delta f}{Q_p} = \eta \cdot R_{th} \cdot TCF \cdot f_0 \qquad (6)$$

where R_{th} is the thermal resistance of the device (R_{th}=1/G_{th}). A crucial parameter that ought to be considered for the design and optimization of the proposed resonant IR detector is the noise equivalent power (NEP), defined as the noise-induced frequency fluctuation f_n divided by the responsivity of the detector:

$$NEP = \frac{f_n}{R_s} = \frac{f_n}{\eta \cdot R_{th} \cdot TCF \cdot f_0} \qquad (7)$$

Among different noise sources, the fundamental limit to frequency stability of a thermal resonant sensor is given by: (1) the thermal fluctuation noise associated with the spontaneous temperature fluctuations of the detector element due to the finite heat conductance G_th to the surroundings, (2) the background fluctuation noise due to radiative heat exchange with the environment, and (3) the thermomechanical noise originated from thermally driven random motion of the mechanical structure. Therefore, the minimization of the NEP associated with each of these three fundamental noise contributions

(respectively NEP_{th}, NEP_{rad}, NEP_{mec}) can be used to drive the design of the IR detector. These NEPs can be expressed by

$$NEP_{th} = \frac{2T_0}{\eta}\sqrt{\frac{K_B}{R_{th}}} \quad NEP_{rad} = \frac{1}{\eta}\sqrt{16A\varepsilon\sigma k_B T_0^5} \quad NEP_{mec} = \sqrt{\frac{K_B T_0}{4P_c}}\frac{1}{\eta \cdot R_{th} \cdot TCF \cdot Q} \qquad (8)$$

where K_B is the Boltzmann constant, T_0 is the temperature of the resonator, A is the area of the device, ε is the emissivity, σ is the Stefan-Boltzman constant, P_c is power used to drive the mechanical resonance in the structure, Q is the resonator's quality factor. The total NEP can be expressed as

$$NEP_{tot} = \sqrt{NEP_{th}^2 + NEP_{rad}^2 + NEP_{mec}^2} \qquad (9)$$

Supplementary Eq. 8 and 9 indicate that minimum NEP, thus a high resolution resonant IR detector, can be achieved by maximizing the device thermal resistance R_{th}, absorption coefficient η, temperature coefficient of frequency, TCF, quality factor Q, and power handling P_c.

Another crucial parameter to be considered is the detector response time, which is limited by the thermal time constantof the device

$$\tau = R_{th} \cdot C_{th} \qquad (10)$$

Supplementary Eq. 8-10 indicate that by improving the thermal isolation of the sensing element from the heat sink (hence increasing R_{th}) the NEP of the device is reduced but its response time is increased. Therefore, a trade-off between these two important performance metrics needs to be generally considered for the design of the detector. Nevertheless, for a given thermal resistance (guaranteeing a satisfactory *NEP*) the response time of the sensor can be reduced by minimizing its thermal capacitance, which directly translates to reducingthe volume of the resonant structure.

Supplementary Note 3. Optimization of the nanoplasmonic piezoelectric metasurface

The spectrally selective plasmonic resonant NEMS structure consists of two functional parts defined by the geometry of the top metal layer: a central section in which a solid top metal plane is employed to effectively confine the electric field induced by the bottom IDT across the piezoelectric nano-plate to achieve efficient piezoelectric transduction of the lateral-extensional mode of vibration in the nano-plate, and two sections in which plasmonic nanostructures are properly patterned in the top metal layer of the structure enabling resonant absorption of IR radiation in the ultrathin piezoelectric vibrating nano-plate (Supplementary Fig. 3). A trade-off between the relative sizes of the two functional parts (i.e. percentages of the nano-plate surface area covered by the plasmonic nanostructures and the solid metal plane) needs to be considered in order to maximize absorption while maintaining sufficiently high electromechanical transduction efficiency (k_t^2~1%). According to this consideration, the design of the nanoplasmonic resonator was optimized by 3D FEM simulations and verified experimentally. The electromechanical coupling coefficient values of nano-plate resonators with different coverages of the top solid metal plate were simulated using 3D COMSOL multiphysics, and compared with the experimentally extracted k_t^2 values of three fabricated nanoplasmonic piezoelectric nano-plate resonators with different partial coverages of the solid metal plate (Supplementary Fig. 4a and b). The three nanoplasmonic resonators were tested in a RF probe station and their electrical admittances versus frequency were measured using a vector network analyzer (as described in the methods section of the main text). The k_t^2values, for all the different configurations, were extracted by MBVD model fitting of the measured admittance amplitude versus frequency curves.

Despite the difference in absolute values due to imperfections in the model and material coefficients used in the simulation, the experimentally recorded variations of k_t^2, for different coverages of the top metal plate, follow the same trend achieved by FEM simulations. The results of this combined FEM analysis and experimental characterization indicate that a coverage of the solid metal plate as low as ~20% (~80% of the nano-plate surface covered by plasmonic nanostructures) is sufficient to maintain a k_t^2~1%. The IR

absorption spectra of the three fabricated nanoplasmonic resonators were also characterized (as described in the Methods section of the main text). The FTIR measurements indicate that high IR absorption (> 80%) can be achieved if the plasmonic nanostructures cover ~ 80% of the nano-plate top surface (Supplementary Fig. 4c and d). Following these results, the coverage of the plasmonic nanostructures on the top metal layer of the plasmonic piezoelectric NEMS resonator prototype discussed in the main text was set to 80% given the demonstrated capability ofachieving high absorption coefficient while maintaining high electromechanical transduction efficiency when this configuration is employed.

Supplementary Note 4. Estimation of the device thermal properties by 3D FEM simulation

The thermal properties of the plasmonic resonant NEMS structure were evaluated by 3D FEM simulation using COMSOL multiphysics. The thermal time constant of the IR detector was simulated by applying an input power of 1 nW (simulating the absorbed IR power by the detector) to the resonator and recording the transient response of the device temperature. Supplementary Fig.5a shows a 1/*e* thermal time constant τ of 5.3 ms. The thermal resistance of the device was calculated by dividing the temperature rise in equilibrium (230 μK) by the input power (1 nW), and found to be 2.3×10^{5} $K\cdot W^{-1}$. The surface temperature profile across the AlN resonator was also simulated, shown in Supplementary Fig. 5b and c. A maximum temperature difference of 15 μK was recorded from the center of the resonator to the anchors, translating to a temperature variation of only ~6.5%. The achievement of such uniform temperature distribution across the AlN resonant nano-plate was enabled by the large thermal resistance of the anchors and relatively low thermal resistance of the AlN nano-plate. The materials properties used in the FEM simulation are listed in Supplementary Fig. 5d.

Supplementary Note 5. Experimental characterization of the noise equivalent power (NEP)

The NEP of the fabricated device was experimentally characterized by measuring all the determining

parameters in Supplementary Eq. 7:

Frequency noise spectral density, f_n: A frequency noise spectral density of f_n~1.46 Hz·Hz$^{-1/2}$ at 100 Hz was extracted from the measurement by monitoring the short term frequency instability. The resonator was excited at a single frequency, f_c = 161.5 MHz, for which the slope of admittance amplitude curve versus frequency is maximum (Fig. 7 in methods in the main text). The peak to peak admittance amplitude fluctuation was recorded (11.7 mdB) and converted to peak to peak frequency fluctuation (96 Hz) by dividing it by the slope (121.3 dB·MHz^{-1}). Then, the root mean square (rms) noise was calculated by dividing the peak to peak frequency fluctuation by 6.6 for a 99.9% confidence, and found to be 14.55 Hz. Finally, the frequency noise spectral density was calculated by dividing the rms frequency noise by the square root of the measurement bandwidth (100 Hz), and found to be f_n~1.46Hz·Hz$^{-1/2}$.

Mechanical resonance frequency, f_0: The resonator admittance versus frequency was measured, as described in the main text, showing a resonance frequency f_0~161.4 MHz (see Fig. 3a in the main text).

Absorption coefficient η: The IR spectral absorptance of the fabricated structure was experimentally characterized, as described in the main text, showing η~80% for an optimized spectral bandwidth around 8.8 μm (see Fig. 2 in the main text).

Temperature coefficient of frequency, *TCF*: the resonator frequency sensitivity to temperature was characterized using a temperature controlled radio frequency (RF) probe station, obtaining TCF~23 ppm·K^{-1} (see Supplementary Fig. 6), which matches the typical TCF values recorded for 500 nm thick AlN contour-mode resonators [5].

Thermal resistance, R_{th}: The device thermal resistance, in air and at room temperature, was directly extracted from the measurement of the admittance amplitude-frequency (A-f) nonlinearity induced by self-heating[6]. The source of admittance *A-f* nonlinearities in AlN contour-mode MEMS resonators is attributed to the softening of the equivalent Young's modulus due to self-heating effects. Therefore, the

thermal resistance of the device can be experimentally extracted by measuring the *A-f* response of the resonator for different input IR powers, according to

$$R_{th} = \frac{\Delta f}{\Delta P_c \cdot (1-\Gamma^2) \cdot k_{IF} \cdot TCF \cdot f_0} \qquad (11)$$

where Δf is resonance frequency shift, ΔP_c is the input RF power to the resonator, $\Gamma = (Z - Z_0)/(Z + Z_0)$ is the reflection coefficient (Z_0 = 50 Ω), k_{IF} is a constant introduced to take into account the effect of the sampling speed of the network analyzer (k_{IF} = 1 for IF bandwidth of 100 Hz used in the measurement), TCF is the temperature coefficient of frequency, f_0 is the resonance frequency of the resonator. The thermal resistances of two devices, with same geometries but different anchors (Detector1 is with Pt anchors and Detector2 is with conventional AlN/Pt anchors) were extracted based on this method. The measured *A-f* response and extracted thermal resistance are shown in Supplementary Fig.7 and listed in Supplementary Table 1, respectively.

The mismatch between the FEM simulated results and the experimentally measured results is attributed to the heat conduction through air, as the experiment was conducted in air and FEM simulation did not include the contribution of heat transfer from air conduction (simulating the case of vacuum). Basically, the measured R_{th} is a parallel combination of the thermal resistance related to the anchors of the resonator and the thermal resistance associated with the air between the resonator and the Silicon substrate, as illustrated in Supplementary Fig. 8. Based on the measured and simulated R_{th}, the R_{Air} of the two devices can be extracted and found to be 5.3 $\times 10^4$ K·W^{-1} and 5.0 $\times 10^4$ K·W^{-1}, respectively. Considering the thermal conductivity of air at 1 atm, and the geometries of the resonator, the average air gap between the resonator and Si substrate can be extracted to be ~20 μm, which is reasonable for a typical XeF_2 isotropic etching of Si to completely release the resonator.

The thermal resistance of the IR detector is limited by the finite thermal resistance of the air gap between the resonator and Si substrate when it is tested in air. Supplementary Fig. 9a shows that the maximum thermal resistance of the IR detector in air at 1 atm (air thermal conductivity of 0.024 Wm^{-1}K^{-1}) is

5.4×10^4K·W^{-1} for a Pt anchor thickness of 10 nm. However, the thermal resistance of the IR detector can be improved significantly by reducing the pressure of air, thus increasing its thermal resistance. Supplementary Fig. 9b shows the calculated thermal resistance in vacuum at 10 mTorr (air thermal conductivity reduced to ~1×10^{-4} Wm^{-1}K^{-1}). It indicates that the total thermal resistance of the IR detector is completely determined by the dimension of the anchor, eliminating the influence of the air conduction. The FEM simulated thermal resistance of 2.3×10^5 K/W matches well with the calculated value (Supplementary Fig. 9b). A maximum thermal resistance of the IR detector is predicted to be 1.7×10^6 K·W^{-1}for a Pt anchor thickness of 10 nm, which could potentially reduce the NEP to the order of ~pW·Hz$^{-1/2}$.

Supplementary References

1 Larson III, J. D., Bradley, R., Wartenberg, S. & Ruby, R. C. in *Ultrasonics Symposium, 2000 IEEE.* 863-868 (IEEE).

2 Rinaldi, M. & Piazza, G. in *Frequency Control and the European Frequency and Time Forum (FCS), 2011 Joint Conference of the IEEE International.* 1-5 (IEEE).

3 Piazza, G., Stephanou, P. J. & Pisano, A. P. Piezoelectric aluminum nitride vibrating contour-mode MEMS resonators. *Microelectromechanical Systems, Journal of* **15**, 1406-1418 (2006).

4 Olsson, R. H., Wojciechowski, K. E., Baker, M. S., Tuck, M. R. & Fleming, J. G. Post-CMOS-compatible aluminum nitride resonant MEMS accelerometers. *Microelectromechanical Systems, Journal of* **18**, 671-678 (2009).

5 Hui, Y. & Rinaldi, M. Fast and high resolution thermal detector based on an aluminum nitride piezoelectric microelectromechanical resonator with an integrated suspended heat absorbing element. *Applied Physics Letters* **102**, 093501, doi:10.1063/1.4794074 (2013).

6 Tazzoli, A., Rinaldi, M. & Piazza, G. Experimental investigation of thermally induced nonlinearities in aluminum nitride contour-mode MEMS resonators. *Electron Device Letters, IEEE* **33**, 724-726 (2012).
